# Externalizing problems in childhood and adolescence predict subsequent educational achievement but for different genetic and environmental reasons

**DOI:** 10.1111/jcpp.12655

**Published:** 2016-11-10

**Authors:** Gary J. Lewis, Kathryn Asbury, Robert Plomin

**Affiliations:** ^1^ Department of PsychologyRoyal HollowayUniversity of LondonEghamSurreyUK; ^2^ Department of EducationUniversity of YorkHeslingtonUK; ^3^ Social, Genetic and Developmental Psychiatry CentreKing's College LondonMRC SocialInstitute of PsychiatryLondonUK

**Keywords:** Education, genetics, longitudinal, twin study, behavior problems, Strengths and Difficulties Questionnaire

## Abstract

**Background:**

Childhood behavior problems predict subsequent educational achievement; however, little research has examined the etiology of these links using a longitudinal twin design. Moreover, it is unknown whether genetic and environmental innovations provide incremental prediction for educational achievement from childhood to adolescence.

**Methods:**

We examined genetic and environmental influences on parental ratings of behavior problems across childhood (age 4) and adolescence (ages 12 and 16) as predictors of educational achievement at age 16 using a longitudinal classical twin design.

**Results:**

Shared‐environmental influences on anxiety, conduct problems, and peer problems at age 4 predicted educational achievement at age 16. Genetic influences on the externalizing behaviors of conduct problems and hyperactivity at age 4 predicted educational achievement at age 16. Moreover, novel genetic and (to a lesser extent) nonshared‐environmental influences acting on conduct problems and hyperactivity emerged at ages 12 and 16, adding to the genetic prediction from age 4.

**Conclusions:**

These findings demonstrate that genetic and shared‐environmental factors underpinning behavior problems in early childhood predict educational achievement in midadolescence. These findings are consistent with the notion that early‐childhood behavior problems reflect the initiation of a life‐course persistent trajectory with concomitant implications for social attainment. However, we also find evidence that genetic and nonshared‐environment innovations acting on behavior problems have implications for subsequent educational achievement, consistent with recent work arguing that adolescence represents a sensitive period for socioaffective development.

## Introduction

The long‐established importance of educational achievement for later‐life success (e.g. Sewell & Hauser, 1975) has led to a significant body of work examining the antecedents of school achievement (Deary, Strand, Smith, & Fernandes, 2007; Fergusson & Horwood, 1998; Shakeshaft et al., 2013; Vitaro, Brendgen, Larose, & Trembaly, 2005). One area of specific focus has been childhood and adolescent behavior problems. Several studies have reported that genetic and environmental factors underpin individual differences in both educational achievement (Asbury & Plomin, 2013; Bartels, Rietveld, Van Baal, & Boomsma, 2002; Shakeshaft et al., 2013) and child and adolescent behavior problems (Eaves et al., 1997; Lewis & Plomin, 2015). Furthermore, these genetic and environmental factors have been found to be moderately to substantially overlapping: that is, some of the same genes and experiences affect both educational achievement and behavior problems (Hicks, Johnson, Iacono, & McGue, 2008; Johnson, McGue, & Iacono, 2005). Less well understood is the extent to which genetic and environmental influences on childhood and adolescent behavior problems predict educational achievement at age 16 (the end of mandatory education in many countries).

In the current study, we used a longitudinal twin design to examine whether childhood and adolescent behavior problems share genetic and environmental influences with educational achievement, and how these influences relate over time. Specifically, we sought to estimate the extent to which genetic and environmental influences acting on behavior problems in early childhood, before formal schooling begins, can predict achievement in public examinations at age 16. Moreover, we examined whether novel genetic and environmental influences on behavior problems, emerging over the course of development (Lewis & Plomin, 2015), would provide additional sources of prediction for educational achievement at age 16. Next we briefly introduce phenotypic and behavioral genetic investigations of educational achievement and behavior problems before moving to formal tests of the role of genetic and environmental influences on behavior problems and educational achievement over childhood and adolescence.

### Behavior problems and educational achievement

Behavior problems pose intuitive risks to the prospects of school success. Children and adolescents with externalizing behavior problems (e.g. conduct problems, hyperactivity) will likely find it harder to pay attention in the classroom or to comply with school rules, and so it would be unsurprising to find inverse associations between externalizing behaviors and school success. A range of studies have examined whether behavior problems and educational achievement are inversely associated and have consistently confirmed this expectation. For example, in a large New Zealand birth cohort, conduct disorder at age 8 was found to predict leaving school at age 18 without educational qualifications (Fergusson & Horwood, 1998). Similarly, in a large Canadian community sample, hyperactivity‐inattention and aggressiveness‐opposition measured in kindergarten were found to predict noncompletion of high school (Vitaro et al., 2005). A large number of other studies also provide support for the link between early‐childhood externalizing behaviors and subsequent low educational achievement (Chen, Huang, Chang, Wang, & Li, 2010; Masten et al., 2005; Veldman et al., 2014).

Beyond externalizing behaviors, the links between educational achievement and behavior problems are more mixed. For example, one study noted that prosociality (often referred to as a behavioral strength: Goodman, 1997) at age 8 predicted educational achievement 5 years later in midadolescence (Caprara, Barbaranelli, Pastorelli, Bandura, & Zimbardo, 2000). A similar observation in a 5‐year longitudinal sample of Chinese school students followed from age 8 also found that prosocial competence predicted academic achievement in subsequent years (Chen et al., 2010). In contrast, other studies have failed to observe prosociality as a predictor of subsequent educational success (e.g. Kokko, Tremblay, Lacourse, Nagin, & Vitaro, 2006). In the domain of internalizing problems, similarly mixed results have been noted. For example, one study reported that higher levels of anxiety in the preschool years were predictive of higher school grades in early adolescence (DiLalla, Marcus, & Wright‐Phillips, 2004) but other research has failed to observe such links (van Lier et al., 2012).

Building on the well‐replicated phenotypic links between externalizing behaviors and educational achievement have been genetically informative studies seeking to assess the relative roles of the genetic and environmental factors underpinning this association. In early childhood, the link between externalizing behavior and educational achievement has been reported to be mostly attributable to shared‐environmental factors, although genetic factors have also been noted to play a role (Newsome, Boisvert, & Wright, 2014). In midchildhood, this pattern appears to shift toward genetic factors accounting for the majority of the phenotypic links between externalizing behaviors and educational achievement. For example, in a large UK cohort [the Twins Early Development Study (TEDS): also used in the current study], hyperactivity and educational success at age 7 were found to share substantial genetic links, alongside more modest nonshared‐environmental links (Saudino & Plomin, 2007). Similarly, results from the Minnesota Twin Family Study showed that at age 11 genetic influences on inattention and educational achievement were highly overlapping, although the genetic link between disruptive behavior and educational achievement, while statistically significant, was less pronounced (Johnson et al., 2005). In the same sample, achievement striving, self‐control, and aggression at age 17 have been reported to be genetically related to educational success (also at age 17), alongside a modest link via nonshared‐environmental influences (Hicks et al., 2008). Finally, work using the TEDS twin cohort reported that standardized UK high‐school exam results at age 16 were heritable and genetically associated with many psychological traits including behavior problems, although associations between educational achievement and specific components of behavior problems were not detailed (Krapohl et al., 2014).

### The current study

These studies provide insights into common genetic and environmental influences underlying observed relationships between behavior problems and educational achievement. However, this literature is still in its infancy and a number of important questions remain unanswered. First, while childhood externalizing behaviors are phenotypically predictive of adolescent educational achievement (Fergusson & Horwood, 1998; Vitaro et al., 2005), are these phenotypic links explained by genetic or environmental factors? Indeed, conduct problems show stable genetic and shared‐environment influences from age 4 to age 16 (Lewis & Plomin, 2015). As such, it is conceivable that either or both of these sources of variance might account for individual differences in their prediction of educational achievement at age 16.

Second, recent work has highlighted that childhood and adolescent externalizing behaviors are underpinned both by stable genetic and environmental influences, as noted above, and also by innovative genetic and environmental influences (i.e. effects that emerge across development: Lewis & Plomin, 2015). As such, do early‐emerging (i.e. ≤age 4) and subsequent (i.e. >age 4) genetic and environmental factors independently relate to later educational achievement?

A range of perspectives have been indirectly informative on this issue. Perhaps most prominently, the developmental taxonomy proposed by Moffitt (1993) stresses that antisocial behavior follows one of two main trajectories: life‐course persistent or adolescent‐limited. The former is argued to reflect disrupted neuropsychological functioning and temperament difficulties, which in turn negatively impact learning and interpersonal relations, and subsequently can serve to impair life outcomes. The latter trajectory is posited to reflect the extreme cases of the otherwise normative adolescent desire to attain status and a distinct personal identity. Here externalizing behaviors are argued to be simply the manifestation of these goals. Of importance, this subset of adolescents is believed to be relatively goal‐directed in their externalizing behaviors: ‘adolescence‐limited delinquents are likely to engage in antisocial behavior in situations where such responses seem profitable to them, but they are also able to abandon antisocial behavior when prosocial styles are more rewarding’ (Moffitt, 1993, p. 686). As such, one would expect that genetic and environmental influences on childhood externalizing would predict educational achievement in adolescence, either as a result of a deleterious developmental cascade, or because the underpinning psychological characteristics of the behavior problems are stable over time and create issues for schooling in a more proximal fashion. In contrast, any genetic and environmental influences on externalizing that emerge in adolescence would be expected to contribute less to the prediction of educational achievement, despite the more proximal nature of these effects. However, recent observations suggest that adolescence is a sensitive period of development for a range of socioaffective processes – such as emotion regulation and impulse control (Steinberg, 2007). These processes have well‐established links to behavior problems (Eisenberg et al., 2001; Silk, Steinberg, & Morris, 2003) and so this sensitive period of socioaffective development may reflect important independent risk factors for subsequent life success in their own right (Blakemore, 2010). As such, it is an open question whether early or later emerging genetic and environmental influences on behavior problems exert the greater impact on educational outcomes.

In addition to our core questions, here we also took the opportunity to examine how internalizing traits (i.e. anxiety, peer problems) and prosociality related to academic achievement, both phenotypically and via underlying genetic and environmental pathways. Establishing the presence and (where relevant) the etiology of such effects is important given the mixed results in these domains, as noted above.

To answer these questions, we used a large and population‐representative sample of UK monozygotic (MZ) and dizygotic (DZ) twins who have been followed since birth as participants in the Twins’ Early Development Study (TEDS; Oliver & Plomin, 2007). TEDS twins have been surveyed on a wide range of behavioral and cognitive characteristics throughout their life span. Here we used the Strengths and Difficulties Questionnaire (SDQ: Goodman, 1997) instrument to assess behavior problems with parental ratings obtained at each age in order to maintain a consistent mode of rating across each wave of measurement. We used SDQ scores at ages 4 and 12 for all SDQ scales, and also for age 16 for prosociality, conduct problems, and hyperactivity (SDQ anxiety and peer problems were not measured at age 16 and so were unavailable for analysis). Educational achievement was measured as the mean score of performance at age 16 in the standardized high‐school completion exams taken in the United Kingdom: the General Certificate of Secondary Education (GCSE).

## Methods

### Participants

Participants were drawn from the TEDS, an ongoing longitudinal study following MZ and DZ twins born in England and Wales between 1994 and 1996 (Haworth, Davis, & Plomin, 2013). The TEDS sample is representative of the UK population (Kovas, Haworth, Dale, & Plomin, 2007) and the project received approval from the Institute of Psychiatry Ethics Committee (05/Q0706/228). Twin zygosity was determined using a parental rating measure of similarity and DNA genotyping (Price et al., 2000). The number of complete twin pairs for each zygosity class across the three measurement points were as follows (also see Table S1, for full details): MZ male pairs: *n* = 720–1,166; MZ female pairs: *n* = 1,028–1,350; DZ male pairs: *n* = 670–1,196; DZ female pairs: *n* = 886–1,247; and DZ opposite sex pairs: *n* = 1,513–2,352. About 52% of those individuals who were assessed at age 4 (for behavior problems) were reassessed at age 16.

### Measures

#### Strength and Difficulties Questionnaire

The SDQ is a short but reliable instrument (25 items: Goodman, 2001; Stone, Otten, Engels, Vermulst, & Janssens, 2010) for measuring psychosocial problems in children (Goodman, 1997). The SDQ consists of five scales measuring anxiety, conduct problems, hyperactivity‐inattention, peer problems, and prosocial behavior. Higher scores indicate greater difficulties (i.e. for anxiety, conduct problems, hyperactivity, peer problems) or strengths (i.e. for prosociality). In the current study, we used parent‐rated scores for the SDQ subscales acquired when the child was 4 and 12 years old. Scores for prosociality, conduct problems, and hyperactivity were also acquired by parental rating when the individual was 16. Cronbach's alpha was low for conduct problems (all ages: *α* range = .52–.57) and for peer problems (age 4: *α *= .47), although in line with previously reported values (Goodman, 2001). Cronbach's alpha was broadly acceptable for the rest of the SDQ measures (anxiety: *α* range = .60–.68; hyperactivity: *α* range = .71–.77; peers age 12: *α* range = .64; prosociality: *α* range = .67–.73).

#### Educational achievement

General Certificate of Secondary Educations are graded from A* (the highest grade) to G (the lowest pass grade). We coded these grades from 11 (A*) to 4 (G): this scoring scheme reflects the fact that sub‐GCSE levels of attainment represent National Curriculum levels 1, 2, and 3, and a G at GCSE was equivalent to Level 4 attainment. We constructed our measure of educational achievement as the mean score for the three required subjects: English (either the English language grade, or the mean of the English language grade and the English literature grade where both exams were taken), Science (the mean of all Science GCSEs taken), and Mathematics.

### Analysis

Correlations between twins differing in their degrees of genetic relatedness (i.e. MZ and DZ twins) are useful as a guiding heuristic to estimate relative magnitudes of genetic and environmental effects (Plomin, DeFries, Knopik, & Neiderhiser, 2013). Three sources of variance are typically estimated using data from MZ and DZ twins: additive genetic (*A*), shared‐environment (*C*), and nonshared‐environment effects (*E*). A reflects the aggregate impact of those genetic effects that sum up to influence a phenotype. *C* reflects the action of environmental factors shared by twins that serve to make them more similar on a particular phenotype. *E* reflects the action of environmental factors unique to individuals within a twin pair that serve to make them differ from each other on a particular phenotype. The presence of genetic effects on a given phenotype is typically inferred if the correlations between MZ twins are larger than the correlations for DZ twins. The presence of shared‐environment effects is inferred if the correlations for DZ twins are larger than half the magnitude of the correlations for the MZ twins. Finally, nonshared‐environmental effects are inferred if correlations for the MZ twins are less than unity. As such, this variance component also contains measurement error.

These correlational analyses were extended using formal model‐fitting of variance–covariance matrices to the twin data. This approach allows parameter estimates in univariate models to be formally tested for significance as well as allowing multivariate models – the core focus of the current study – to be analyzed. The validity of inferences from twin analyses, as with all methods, rest on certain assumptions. First, that MZ and DZ twins are equally correlated with regard to environments of etiological importance for the trait under study (i.e. the equal‐environments assumption); second, the absence of parental assortative mating for the trait under study. Violations of the former will serve to (spuriously) increase estimates of heritability, whereas violation of the latter will serve to (spuriously) increase estimates of shared‐environment effects.

**Figure 1:**
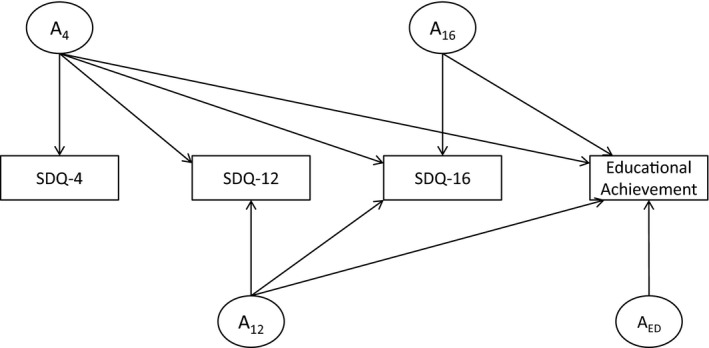
Graphical representation of the longitudinal Cholesky decomposition for Strengths and Difficulties Questionnaire (SDQ) traits and educational achievement. *A* = additive genetic influences; shared‐ and nonshared‐environmental influences were also modeled, and took the same form as the *A* pathways (i.e. *C*_4_, *C*_12_, *C*_16_, *E*_4_, *E*_12_, and *E*_16_), but are omitted here in the interests of visual clarity; SDQ‐16 was only available for conduct, hyperactivity, and prosociality

In the current study, longitudinal analyses were central to our tests: We sought to estimate the extent to which genetic and environmental effects underlying SDQ measures at ages 4, 12, and 16 (for conduct problems, hyperactivity, and prosociality only) were associated with educational achievement at age 16. To perform this analysis, we used the Cholesky decomposition. The Cholesky decomposition specifies as many factors as there are variables for each source of variance, with each subsequent factor having one fewer pathway than the preceding factor (see Figure 1). In other words, for additive genetic effects (*A*), the first latent factor loads on all of the *n* measured variables: The subsequent latent factors load on *n*−1, *n*−2…*n*−*i* variables. In this way, each factor accounts for as much of the remaining variance as possible, until the last factor accounts for just the residual variance in the last measured variable. This is repeated for the shared‐environment factors (*C*) and nonshared‐environmental factors (*E*). This design makes it possible to estimate the extent to which early‐emerging genetic and environmental influences on an SDQ trait predict later educational achievement. Moreover, this design allows us to examine whether innovative genetic and environmental factors – that is sources of variance independent of earlier genetic and environmental influences – contribute additional genetic and environmental prediction to educational achievement at age 16. Twin models were fitted using full‐information maximum‐likelihood in OpenMx 2.0 (Boker et al., 2011) running within R 3.2 (R Development Core Team, 2015).

## Results

Descriptive statistics for all of the study variables are detailed in full in Table 1. Assumption testing using all twins indicated that means and variances could be equated across twin order, zygosity, and sex for most variables, with the small number of significant differences observed consistent with the large number of tests performed. Of note, however, was evidence for modest‐to‐moderate mean sex differences, particularly for hyperactivity and prosociality. Sex‐limitation modeling (testing for quantitative and qualitative genetic and environmental differences across sex) largely indicated that genetic and environmental influences could be equated across sex, with the significant differences that were observed mostly being either small in magnitude or, again, consistent with the large number of tests performed. Following these observations, we pooled our sample across sex, but used sex‐residualized variables for all twin analyses.

**Table 1:** Descriptive statistics for SDQ subscales and educational achievement

Measure	*α*	MZm *M* (*SD*)	MZf *M* (*SD*)	DZm *M* (*SD*)	DZf *M* (*SD*)	DZosm *M* (*SD*)	DZosf *M* (*SD*)
SDQ (age 4)							
Anxiety	.60	1.28 (1.35)	1.41 (1.43)	1.39 (1.44)	1.49 (1.49)	1.33 (1.40)	1.32 (1.43)
Conduct	.54	2.26 (1.58)	1.91 (1.46)	2.27 (1.60)	1.97 (1.54)	2.16 (1.60)	1.88 (1.50)
Hyperactivity	.76	4.37 (2.24)	3.74 (2.07)	4.21 (2.44)	3.77 (2.36)	4.38 (2.36)	3.32 (2.18)
Peer problems	.47	1.40 (1.41)	1.23 (1.34)	1.70 (1.57)	1.45 (1.48)	1.63 (1.57)	1.37 (1.42)
Prosociality	.69	7.04 (1.84)	7.56 (1.78)	7.11 (1.90)	7.61 (1.81)	7.09 (1.92)	7.71 (1.77)
SDQ (age 12)							
Anxiety	.68	1.65 (1.80)	1.92 (1.97)	1.65 (1.84)	1.93 (1.94)	1.65 (1.86)	1.89 (1.97)
Conduct	.57	1.43 (1.45)	1.16 (1.34)	1.49 (1.56)	1.21 (1.41)	1.40 (1.53)	1.23 (1.40)
Hyperactivity	.77	3.35 (2.25)	2.29 (1.96)	3.23 (2.39)	2.50 (2.13)	3.48 (2.49)	2.14 (1.87)
Peer problems	.64	1.11 (1.50)	0.88 (1.28)	1.23 (1.64)	1.04 (1.44)	1.27 (1.66)	0.96 (1.36)
Prosociality	.67	8.31 (1.72)	8.83 (1.50)	8.20 (1.74)	8.84 (1.49)	8.28 (1.73)	8.78 (1.49)
SDQ (age 16)							
Conduct	.52	1.16 (1.31)	1.12 (1.28)	1.25 (1.34)	1.23 (1.43)	1.26 (1.45)	1.16 (1.35)
Hyperactivity	.71	2.45 (2.01)	1.88 (1.65)	2.53 (2.03)	2.06 (1.99)	2.78 (2.19)	1.84 (1.71)
Prosociality	.73	8.00 (1.95)	8.53 (1.86)	7.91 (2.01)	8.56 (1.78)	7.93 (2.01)	8.46 (1.81)
Educational achievement	–	8.75 (1.20)	8.99 (1.14)	8.81 (1.16)	8.99 (1.18)	8.73 (1.22)	9.03 (1.14)

*M*, mean (*SD*: standard deviation); *α*, Cronbach's alpha for scale scores collapsed across sex and zygosity; MZ, monozygotic; DZ, dizygotic; m, male; f, female; os, opposite sex; SDQ, Strengths and Difficulties Questionnaire.

Higher scores indicate greater difficulties (i.e. anxiety, conduct problems, hyperactivity, peer problems) or strengths (i.e. prosociality).

### Phenotypic analyses

We first examined whether age 4 SDQ traits predicted educational achievement at age 16. Correlational analyses showed significant negative links with anxiety (*r* = −.06, *p* < .001), conduct problems (*r* = −.19, *p* < .001), hyperactivity (*r* = −.23, *p* <.001), and peer problems (*r* = −.09, *p* < .001). No association between age 4 prosociality and educational achievement was observed (*r* = .02, *p* > .05). These associations remained significant when controlling for parental socioeconomic status (indexed by parental education level, occupation, and family income) and sex. The full set of intercorrelations is presented in Table S2.

**Figure 2:**
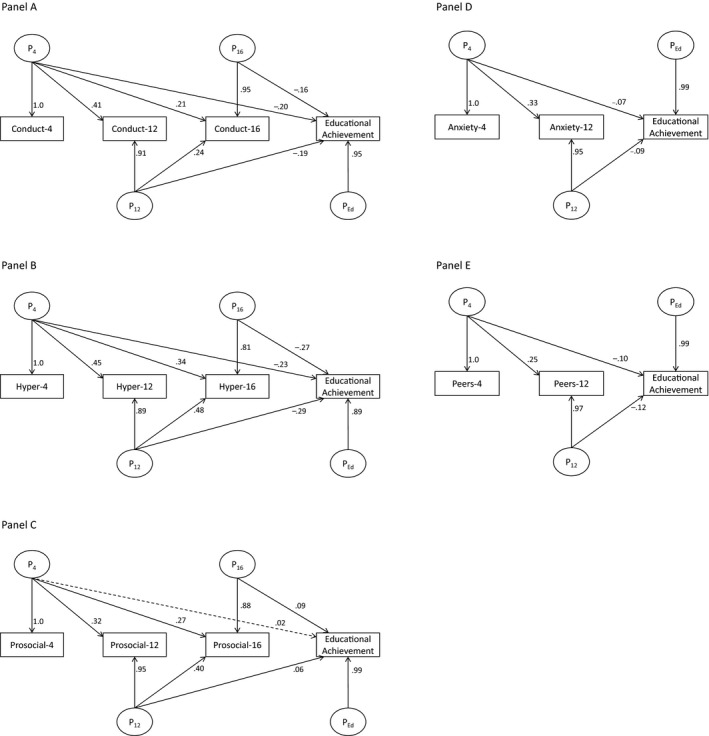
Phenotypic Cholesky decomposition modeling results for Strengths and Difficulties Questionnaire (SDQ) traits and educational achievement. Bolded lines = *p* < .05; P = phenotypic effects; 4/12/16 = age 4/12/16; values are standardized path loadings. (A) Details the model for conduct problems and educational achievement; (B) details the model for hyperactivity and educational achievement; (C) details the model for prosociality and educational achievement; (D) details the model for anxiety and educational achievement; and (E) details the model for peer problems and educational achievement

We next sought to test whether behavior problems at age 12 and 16 added to this prediction. As such we built a series of phenotypic Cholesky decomposition models (see Figure 2), which followed the same logic as detailed above for the twin analyses. These analyses indicated that all SDQ traits at age 4 – with the exception of prosociality – were significant predictors of age 16 educational achievement (in line with the correlational analyses reported above). Of importance, we also observed that in all cases, SDQ traits provided incremental prediction at subsequent ages. The size of these effects ranged from moderate (hyperactivity_age12_ → educational achievement *β *= −.29) to modest (prosociality_age12_ → educational achievement *β *= .06; see Figure 2). Controlling for parental socioeconomic status and sex did not lead to any notable changes in the magnitude or significance of path estimates.

### Twin analyses

Educational achievement showed significant genetic (*A* = .55, *p* < .001), shared‐environment (*C* = .35, *p* < .001), and nonshared‐environment (*E* = .11, *p* < .001) effects (also see Shakeshaft et al., 2013). The univariate twin analyses for the behavioral problems variables have been reported in other published work (Lewis, Haworth, & Plomin, 2014; Saudino & Plomin, 2007; Shakeshaft et al., 2013) and so are not detailed in full here (but see Tables S3–S5). In brief, though, SDQ traits were all underpinned by moderate‐to‐large genetic and nonshared‐environmental influences, with modest shared‐environmental influences evident for SDQ‐conduct problems and SDQ‐prosociality.

**Figure 3:**
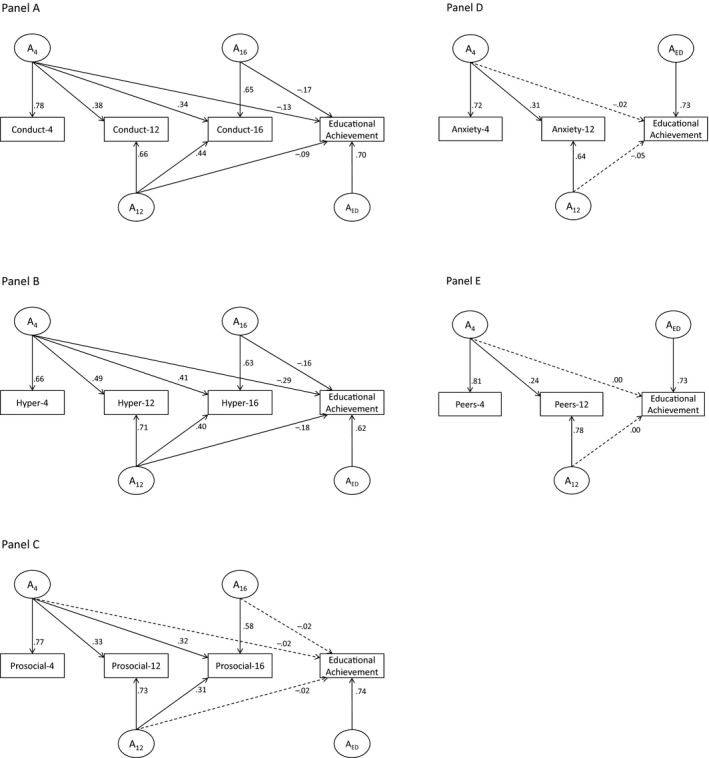
Longitudinal additive genetic modeling results for Strengths and Difficulties Questionnaire (SDQ) traits and educational achievement. Bolded lines = *p* < .05; *A* = additive genetic effects; 4/12/16 = age 4/12/16; values are standardized path loadings. (A) Details the model for conduct problems and educational achievement; (B) details the model for hyperactivity and educational achievement; (C) details the model for prosociality and educational achievement; (D) details the model for anxiety and educational achievement; and (E) details the model for peer problems and educational achievement

**Figure 4:**
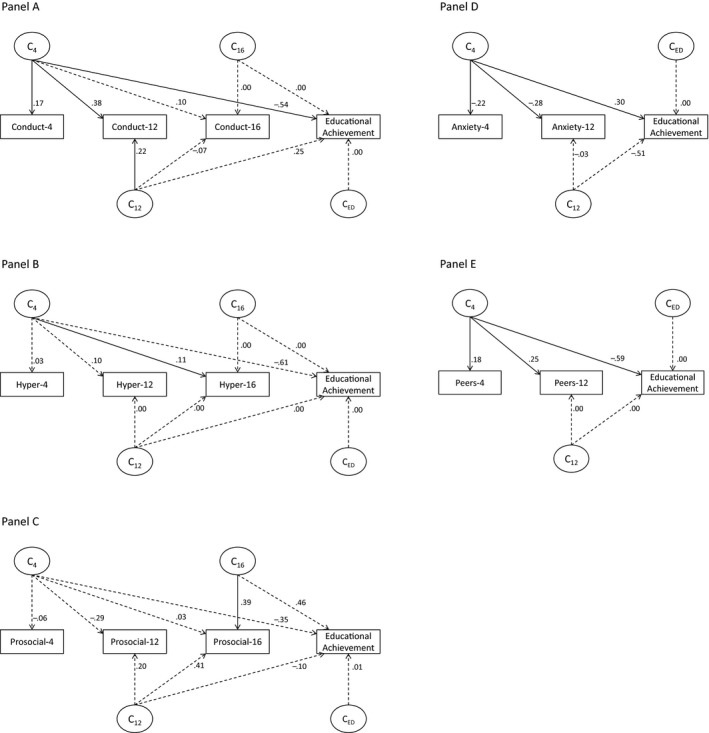
Longitudinal shared‐environment modeling results for Strengths and Difficulties Questionnaire (SDQ) traits and educational achievement. Bolded lines = *p* < .05; *C* = shared‐environment effects; 4/12/16 = age 4/12/16; values are standardized path loadings. (A) Details the model for conduct problems and educational achievement; (B) details the model for hyperactivity and educational achievement; (C) details the model for prosociality and educational achievement; (D) details the model for anxiety and educational achievement; and (E) details the model for peer problems and educational achievement

**Figure 5:**
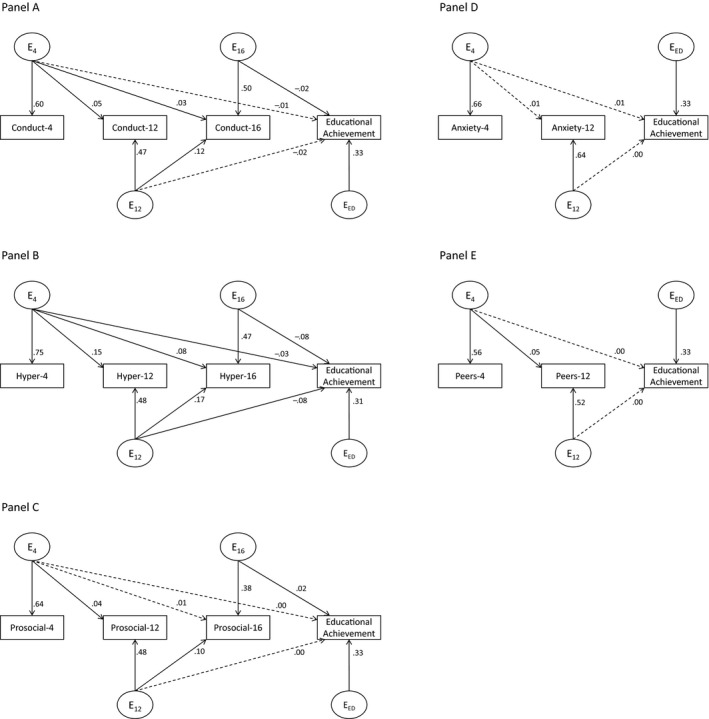
Longitudinal nonshared‐environment modeling results for Strengths and Difficulties Questionnaire (SDQ) traits and educational achievement. Bolded lines = *p* < .05; *E* = nonshared‐environment effects; 4/12/16 = age 4/12/16; values are standardized path loadings. (A) Details the model for conduct problems and educational achievement; (B) details the model for hyperactivity and educational achievement; (C) details the model for prosociality and educational achievement; (D) details the model for anxiety and educational achievement; and (E) details the model for peer problems and educational achievement

We next turned to tests of genetic and environmental influences underpinning the phenotypic associations between SDQ traits and educational achievement. We built a series of Cholesky models with SDQ traits at age 4, age 12, and age 16 (conduct problems, hyperactivity, and prosociality only), and educational achievement score entered in chronological order from left to right (see Figure 1 or Figures 3, 4, 5). To test whether SDQ traits were genetically and environmentally associated with educational achievement, we examined each of the genetic paths shared between educational achievement and SDQ age 4, age 12, and age 16, respectively. These parameters correspond to *A*_4_, *A*_12_, and *A*_16_ to educational achievement in Figure 1. For conduct problems, these genetic paths were significant at all ages: conduct_A4_‐educational achievement: Δ*χ* ^2^(1) =17.88, *p* < .001; conduct_A12_‐educational achievement: Δ*χ* ^2^(1) = 6.03, *p* = .01; conduct_A16_‐educational achievement: Δ*χ* ^2^(1) = 20.67, *p* = <.001. Similar results were observed for hyperactivity: hyperactivity_A4_‐educational achievement: Δ*χ* ^2^(1) = 98.85, *p* < .001; hyperactivity_A12_‐educational achievement: Δ*χ* ^2^(1) = 27.22, *p* < .001; hyperactivity_A16_‐educational achievement: Δ*χ* ^2^(1) = 22.59, *p* = <.001. No genetic associations were observed between the other three SDQ traits (at any age) and educational achievement: all Δ*χ* ^2^(1) < 1.21, all *p* > .27.

Shared‐ and nonshared‐environmental influences were examined using the same principles detailed above. For anxiety, conduct problems, and peer problems, shared‐environmental influences were significantly associated with educational achievement, and these influences were all reflective of early‐emerging shared‐environment influences with broadly stable effects thereafter: anxiety_C4_‐educational achievement: Δ*χ* ^2^(1) = 7.81, *p* = .005; conduct_C4_‐educational achievement: Δ*χ* ^2^(1) = 14.56, *p* < .001; peer problems_C4_‐educational achievement: Δ*χ* ^2^(1) = 19.91, *p* < .001. No further shared‐environmental associations were observed between SDQ traits (at any age) and educational achievement.

Nonshared‐environmental influences were more nuanced. For conduct problems, these paths were significant at ages 12 (Δ*χ* ^2^(1) = 4.80, *p* = .03) and 16 (Δ*χ* ^2^(1) = 7.44, *p* = .006); and for hyperactivity, at ages 4 (Δ*χ* ^2^(1) = 10.04, *p* = .002), 12 (Δ*χ* ^2^(1) = 83.08, *p* < .001), and 16 (Δ*χ* ^2^(1) = 95.30, *p* < .001). For prosociality, these overlapping influences were only apparent cross‐sectionally at age 16 (Δ*χ* ^2^(1) = 7.07, *p* = .008).

Finally, we examined the magnitude of the overlap between genetic and environmental influences on SDQ traits and educational achievement. Genetic influences on conduct problems that were present by age 4 accounted for 3.1% of the genetic effects underpinning educational achievement at age 16. Genetic influences on conduct problems that were present by ages 12 and 16 accounted for a further 1.5% and 5.3%, respectively. Genetic influences on hyperactivity that were present by age 4 accounted for 16% of the genetic effects underpinning educational achievement at age 16. Genetic influences on hyperactivity that were present by ages 12 and 16 accounted for a further 6.2% and 4.9%, respectively. Shared‐environmental influences on peer problems fully overlapped (100%) with the shared‐environment influences on educational achievement, and these overlapping influences were present from age 4. Shared‐environmental influences on anxiety accounted for 25.7% of the shared‐environment influences on educational achievement, and these overlapping influences were present from age 4. Nonshared‐environmental influences on hyperactivity that were present by age 4 accounted for 1% of the nonshared‐environmental effects underpinning educational achievement at age 16. Nonshared‐environmental influences on hyperactivity that were present by ages 12 and 16 accounted for a further 5.8% and 5.8%, respectively. Conduct problems and prosociality at age 16 each showed nonshared‐environmental influences that overlapped with educational achievement: <1% in both cases. Full model parameter estimates for additive genetic, shared‐, and nonshared‐environmental influences are detailed in Figures 3, 4, 5.

### Subsidiary analyses

The above analyses independently addressed each of the behavioral problems and their respective links to educational achievement. Our results indicated that conduct problems and hyperactivity are both genetically linked with educational achievement. This observation gives rise to the question of whether the genetic contribution from conduct problems to educational achievement is specific to conduct problems, or overlaps with hyperactivity. Similarly, conduct problems, anxiety, and peer problems all showed shared‐environment links with educational achievement. As such, is the shared‐environmental contribution from conduct problems to educational achievement specific to conduct problems, or does it overlap with that of anxiety and peer problems? The Cholesky decomposition is ill‐suited to address this issue as the general factor also necessarily includes specific variance to whichever variable is included first in the model. As such we used the independent pathways model (see Figure S1). This model specifies both a general factor and specific factors for genetic, shared‐, and nonshared‐environment effects. Accordingly, if this model shows a good fit to the data (relative to the baseline Cholesky), it provides evidence that genetic and environmental covariance between the measured variables can be accounted for by the general factor. For these analyses, we focused specifically on age 4 behavioral problems as not all measures were available at age 16.

We first used this model to examine whether the genetic influences underpinning conduct problems and hyperactivity provide distinct or common genetic prediction for educational achievement. The independent pathways model provided a good fit to the data and was not appreciably different (Burnham & Anderson, 2004) to the Cholesky decomposition (AIC_Cholesky_ = 15333.01 vs. AIC_IPMod_ = 15335.40). We detail the parameter estimates of the independent pathways model in Figure S1. As such, this analysis indicates that while conduct problems and hyperactivity (at age 4) are both genetic predictors of age 16 educational achievement, this prediction reflects a common etiology.

We next examined whether the shared‐environment links between behavioral problems – specifically, conduct problems, anxiety, and peer problems – and educational achievement reflected processes specific to each behavioral problem or a more general etiology. The independent pathways model fitted substantially less well (AIC_Cholesky_ = 21546.35 vs. AIC_IP_ = 21561.16); however, this result may simply reflect the omission of a specific genetic effect common to conduct problems and educational achievement; that is the general genetic factor forces any link between age 4 conduct problems and educational achievement to also explain genetic influences on peer problems and anxiety. Indeed, a modified independent pathway model including this parameter provided a more parsimonious fit to the data than the Cholesky (AIC_Cholesky_ = 21546.35 vs. AIC_IPMod_ = 21539.22: see Figure S2). In aggregate, this set of analyses indicates that while conduct problems, anxiety, and peer problems (at age 4) are all shared‐environment predictors of age 16 educational achievement, these environmental factors reflect generalized rather than specific sources of prediction.

## Discussion

The current study examined the association between child and adolescent behavior problems and educational achievement at age 16. At the phenotypic level, anxiety, conduct problems, hyperactivity, and peer problems (as rated by parents) at age 4 all predicted lower levels of educational achievement at age 16, although the magnitude of these predictions was modest for anxiety and peer problems. For each of these variables, incremental prediction for educational achievement was observed at the subsequent measurement points. Prosociality positively predicted educational achievement from age 12, with incremental prediction at age 16. These results support previous findings reporting early‐childhood links from externalizing to school success (Fergusson & Horwood, 1998; Vitaro et al., 2005), as well as help to clarify the role of internalizing behaviors and prosociality on educational achievement in light of mixed findings in the literature (Caprara et al., 2000; DiLalla et al., 2004; Kokko et al., 2006; van Lier et al., 2012).

The etiology (i.e. genetic and environmental underpinnings) of the association for the links between early‐childhood behavior problems and later educational achievement was largely specific to each of the behavior problems. The link between early‐childhood conduct problems and later educational achievement was explained by genetic and shared‐environmental factors. The link between early‐childhood hyperactivity and later educational achievement was also explained in part by genetic factors, but here nonshared‐environmental factors accounted for the remainder of the association. This result is notable in light of the rarity of nonshared‐environmental stability over time (e.g. Burt, Klahr, & Klump, 2015; Turkheimer & Waldron, 2000; although see Livingstone et al., 2016). The link between early‐childhood anxiety and peer problems and later educational achievement was explained by shared‐environmental factors. Of note, the genetic influences linking conduct problems and hyperactivity with educational achievement were themselves substantially overlapping. Similarly, the shared‐environmental contribution from conduct problems to educational achievement overlapped substantially with that of anxiety and peer problems. These findings, particularly the observations for the externalizing problems, are consistent with the notion that early‐emerging behavior problems reflect long‐standing challenges to life outcomes (Moffitt, 1993), here exemplified by the important life variable of educational achievement.

For both conduct problems and hyperactivity, we also found evidence for genetic and nonshared‐environment innovations that emerged at ages 12 and 16 and provided incremental prediction for educational achievement. These results are consistent (particularly in the context of genetic innovation) with the notion of adolescence as a sensitive period of socioaffective development with implications in turn for educational outcomes (Blakemore, 2010). In contrast, the associations from anxiety and peer problems to educational achievement did not show genetic or environmental innovations, instead being wholly accounted for by early‐emerging shared‐environment effects.

These findings raise some intriguing questions. First, what processes might explain the shared‐environmental influences acting on age 4 conduct problems, anxiety, and peer problems, which in turn impact on later educational achievement? Some possible factors might include parental warmth/support (Roelofs, Meesters, ter Huurne, Bamelis, & Muris, 2006), parental control (Barber, 1996), or family chaos (Hanscombe, Haworth, Davis, Jaffee, & Plomin, 2011). Low levels of parental concern for the welfare and outcomes of the child might plausibly manifest in conduct problems, anxiety, and peer problems, and in turn impact educational outcomes, either directly – for example through limited shared book reading or interaction – or indirectly – for example as a consequence of behavior problems impairing learning opportunities. Broader shared experiences beyond the home – such as preschool quality or neighborhood‐level deprivation (Caspi, Taylor, Moffitt, & Plomin, 2000; Reijneveld, Brugman, Verhulst, & Verloove‐Vanhorick, 2005) – might similarly explain this pattern of shared‐environmental effects.

Second, what processes might explain the genetic influences common between externalizing problems (i.e. conduct problems and hyperactivity) and educational achievement? One possibility is individual differences in executive functioning, particularly in the context of emotion and impulse management. Such mechanisms are likely to have direct effects on the expression of externalizing behaviors (Barkley, 1997; Moffitt, 1993; Morgan & Lilienfeld, 2000) and may also impair educational development through failures to persevere when the workload becomes difficult, or indirectly as a consequence of exclusion from class activities due to poor behavior.

A number of limitations require discussion. First, the classical twin design is subject to a number of assumptions, such as the equal‐environments assumption (Plomin et al., 2013). Future studies that can capitalize on additional family structures in order to provide more assumption‐free estimates would be valuable, although it is noteworthy that research testing whether violations of the equal‐environments assumption are apparent for psychopathology has found little evidence for this potential source of bias (Kendler, Neale, Kessler, Heath, & Eaves, 1993). Second, with the current study design, we cannot draw inferences concerning the genetic and environmental mechanisms underpinning the observed links between early‐childhood behavioral problems and later educational achievement. For instance, these traits may exert their influence on subsequent school success through the initiation of a deleterious developmental cascade (i.e. bad school behavior early on leads to poor skill development, with the associated knock‐on effects for subsequent intellectual development) or because of stable influences that act more proximately. Third, although Cronbach's alpha was consistent with previous work (e.g. Goodman, 2001), these values fell below conventional standards for conduct problems (all ages) and for peer problems (age 4). It is noteworthy, however, that some debate exists over whether modest Cronbach's alpha values signal need for concern. If one uses a broad content coverage and quickly administrable instrument with just a few items per scale, as is the case with the SDQ instrument, one should expect, and perhaps even desire, such ‘modest’ alphas (e.g. Boyle, 1991).

In summary, in the current study, we have shown that genetic, shared‐environmental, and (to a lesser extent) nonshared‐environmental influences on behavior problems in early childhood are predictive of educational achievement in major public examinations at age 16, consistent with work emphasizing life‐course persistence of behavior problems and the concomitant negative life outcomes. Of importance, we also observed that new genetic and nonshared‐environmental influences – that is, genetic influences on conduct problems and hyperactivity emerging during childhood and adolescent development – were also predictive of educational achievement at 16, consistent with the notion that adolescence represents a sensitive period for socioaffective development.

**Key points**

- Previous work shows childhood behavior problems predict subsequent educational achievement.
- However, it is unclear whether these effects represent genetic or environmental factors.
- To address this issue, we used a longitudinal classic twin design: behavior problems were assessed at ages 4, 12, and 16; educational achievement was assessed at age 16.
- Shared‐environmental influences on anxiety, conduct problems, and peer problems at age 4 predicted educational achievement at age 16.
- Genetic influences on conduct problems and hyperactivity at age 4 predicted educational achievement at age 16.
- Novel genetic and nonshared‐environmental influences acting on conduct problems and hyperactivity emerged at ages 12 and 16, adding to the genetic prediction from age 4.

## Supporting information


**Figure S1.** Independent pathway model for conduct problems (age 4), hyperactivity (age 4), and educational achievement (age 16).
**Figure S2.** Modified independent pathway model for peer problems (age 4), anxiety (age 4), conduct problems (age 4), and educational achievement (age 16).
**Table S1.** Number of complete twin pairs across zygosity and sex for all study measures.
**Table S2.** Phenotypic correlations between SDQ variables and educational achievement.
**Table S3.** Cross‐sectional twin analysis results for SDQ variables.
**Table S4.** Correlations between SDQ variables and educational achievement for MZ twin pairs.
**Table S5.** Correlations between SDQ variables and educational achievement for DZ twin pairs.

## References

[jcpp12655-bib-0500] Asbury, K. , & Plomin, R. (2013). G is for genes: The impact of genetics on education. Oxford, UK: Wiley‐Blackwell.

[jcpp12655-bib-0001] Barber, B.K. (1996). Parental psychological control: Revisiting a neglected construct. Child Development, 67, 3296–3319. 9071782

[jcpp12655-bib-0002] Barkley, R.A. (1997). Behavioral inhibition, sustained attention, and executive functions: Constructing a unifying theory of ADHD. Psychological Bulletin, 121, 65–94. 9000892 10.1037/0033-2909.121.1.65

[jcpp12655-bib-0003] Bartels, M. , Rietveld, M.J. , Van Baal, G.C.M. , & Boomsma, D.I. (2002). Heritability of educational achievement in 12‐year‐olds and the overlap with cognitive ability. Twin Research, 5, 544–553. 12573186 10.1375/136905202762342017

[jcpp12655-bib-0004] Blakemore, S.J. (2010). The developing social brain: Implications for education. Neuron, 65, 744–747. 20346751 10.1016/j.neuron.2010.03.004 PMC2860242

[jcpp12655-bib-0006] Boker, S. , Neale, M. , Maes, H. , Wilde, M. , Spiegel, M. , Brick, T. , … & Mehta, P. (2011). OpenMx: An open source extended structural equation modeling framework. Psychometrika, 76, 306–317. 23258944 10.1007/s11336-010-9200-6 PMC3525063

[jcpp12655-bib-0007] Boyle, G.J. (1991). Does item homogeneity indicate internal consistency or item redundancy in psychometric scales? Personality and Individual Differences, 12, 291–294.

[jcpp12655-bib-0008] Burnham, K.P. , & Anderson, D.R. (2004). Multimodel inference understanding AIC and BIC in model selection. Sociological Methods and Research, 33, 261–304.

[jcpp12655-bib-0009] Burt, S.A. , Klahr, A.M. , & Klump, K.L. (2015). Do non‐shared environmental influences persist over time? An examination of days and minutes. Behavior Genetics, 45, 24–34. 25262214 10.1007/s10519-014-9682-6 PMC4289645

[jcpp12655-bib-0010] Caprara, G.V. , Barbaranelli, C. , Pastorelli, C. , Bandura, A. , & Zimbardo, P.G. (2000). Prosocial foundations of children's academic achievement. Psychological Science, 11, 302–306. 11273389 10.1111/1467-9280.00260

[jcpp12655-bib-0011] Caspi, A. , Taylor, A. , Moffitt, T.E. , & Plomin, R. (2000). Neighborhood deprivation affects children's mental health: Environmental risks identified in a genetic design. Psychological Science, 11, 338–342. 11273396 10.1111/1467-9280.00267

[jcpp12655-bib-0012] Chen, X. , Huang, X. , Chang, L. , Wang, L. , & Li, D. (2010). Aggression, social competence, and academic achievement in Chinese children: A 5‐year longitudinal study. Development and Psychopathology, 22, 583–592. 20576180 10.1017/S0954579410000295

[jcpp12655-bib-0013] Deary, I.J. , Strand, S. , Smith, P. , & Fernandes, C. (2007). Intelligence and educational achievement. Intelligence, 35, 13–21.

[jcpp12655-bib-0014] DiLalla, L.F. , Marcus, J.L. , & Wright‐Phillips, M.V. (2004). Longitudinal effects of preschool behavioral styles on early adolescent school performance. Journal of School Psychology, 42, 385–401.

[jcpp12655-bib-0015] Eaves, L.J. , Silberg, J.L. , Meyer, J.M. , Maes, H.H. , Simonoff, E. , Pickles, A. , … & Hewitt, J.K. (1997). Genetics and developmental psychopathology: 2. The main effects of genes and environment on behavioral problems in the Virginia Twin Study of Adolescent Behavioral Development. Journal of Child Psychology and Psychiatry, 38, 965–980. 9413795 10.1111/j.1469-7610.1997.tb01614.x

[jcpp12655-bib-0016] Eisenberg, N. , Cumberland, A. , Spinrad, T.L. , Fabes, R.A. , Shepard, S.A. , Reiser, M. , … & Guthrie, I.K. (2001). The relations of regulation and emotionality to children's externalizing and internalizing problem behavior. Child Development, 72, 1112–1134. 11480937 10.1111/1467-8624.00337

[jcpp12655-bib-0017] Fergusson, D.M. , & Horwood, L.J. (1998). Early conduct problems and later life opportunities. Journal of Child Psychology and Psychiatry, 39, 1097–1108. 9844980

[jcpp12655-bib-0018] Goodman, R. (1997). The Strengths and Difficulties Questionnaire: A research note. Journal of Child Psychology and Psychiatry, 38, 581–586. 9255702 10.1111/j.1469-7610.1997.tb01545.x

[jcpp12655-bib-0019] Goodman, R. (2001). Psychometric properties of the Strengths and Difficulties Questionnaire (SDQ). Journal of the American Academy of Child and Adolescent Psychiatry, 40, 1337–1345. 11699809 10.1097/00004583-200111000-00015

[jcpp12655-bib-0020] Hanscombe, K.B. , Haworth, C. , Davis, O.S. , Jaffee, S.R. , & Plomin, R. (2011). Chaotic homes and school achievement: A twin study. Journal of Child Psychology and Psychiatry, 52, 1212–1220. 21675992 10.1111/j.1469-7610.2011.02421.x PMC3175268

[jcpp12655-bib-0501] Haworth, C.M. , Davis, O.S. , & Plomin, R. (2013). Twins Early Development Study (TEDS): A genetically sensitive investigation of cognitive and behavioral development from childhood to young adulthood. Twin Research and Human Genetics, 16, 117–125. 23110994 10.1017/thg.2012.91 PMC3817931

[jcpp12655-bib-0021] Hicks, B.M. , Johnson, W. , Iacono, W.G. , & McGue, M. (2008). Moderating effects of personality on the genetic and environmental influences of school grades helps to explain sex differences in scholastic achievement. European Journal of Personality, 22, 247–268. 20228967 10.1002/per.671 PMC2836730

[jcpp12655-bib-0022] Johnson, W. , McGue, M. , & Iacono, W.G. (2005). Disruptive behavior and school grades: Genetic and environmental relations in 11‐year‐olds. Journal of Educational Psychology, 97, 391–405.

[jcpp12655-bib-0023] Kendler, K.S. , Neale, M.C. , Kessler, R.C. , Heath, A.C. , & Eaves, L.J. (1993). A test of the equal‐environment assumption in twin studies of psychiatric illness. Behavior Genetics, 23, 21–27. 8476388 10.1007/BF01067551

[jcpp12655-bib-0024] Kokko, K. , Tremblay, R.E. , Lacourse, E. , Nagin, D.S. , & Vitaro, F. (2006). Trajectories of prosocial behavior and physical aggression in middle childhood: Links to adolescent school dropout and physical violence. Journal of Research on Adolescence, 16, 403–428.

[jcpp12655-bib-0025] Kovas, Y. , Haworth, C.M.A. , Dale, P.S. , & Plomin, R. (2007). The genetic and environmental origins of learning abilities and disabilities in the early school years. Monographs of the Society for Research in Child Development, 72, vii–160. 10.1111/j.1540-5834.2007.00439.x PMC2784897 17995572

[jcpp12655-bib-0026] Krapohl, E. , Rimfeld, K. , Shakeshaft, N.G. , Trzaskowski, M. , McMillan, A. , Pingault, J.B. , … & Plomin, R. (2014). The high heritability of educational achievement reflects many genetically influenced traits, not just intelligence. Proceedings of the National Academy of Sciences of the USA, 111, 15273–15278. 25288728 10.1073/pnas.1408777111 PMC4210287

[jcpp12655-bib-0027] Lewis, G.J. , Haworth, C.M.A. , & Plomin, R. (2014). Identical genetic influences underpin behavior problems in adolescence and basic traits of personality. Journal of Child Psychology and Psychiatry, 55, 865–875. 24256444 10.1111/jcpp.12156 PMC4282475

[jcpp12655-bib-0028] Lewis, G.J. , & Plomin, R. (2015). Heritable influences on behavioural problems from early childhood to mid‐adolescence: Evidence for genetic stability and innovation. Psychological Medicine, 45, 2171–2179. 25765219 10.1017/S0033291715000173 PMC4462158

[jcpp12655-bib-0029] Livingstone, L.T. , Coventry, W.L. , Corley, R.P. , Willcutt, E.G. , Samuelsson, S. , Olson, R.K. , & Byrne, B. (2016). Does the environment have an enduring effect on ADHD? A longitudinal study of monozygotic twin differences in children. Journal of Abnormal Child Psychology. doi:10.1007/s10802‐016‐0145‐9 10.1007/s10802-016-0145-9 PMC5027180 26993487

[jcpp12655-bib-0030] Masten, A.S. , Roisman, G.I. , Long, J.D. , Burt, K.B. , Obradović, J. , Riley, J.R. , … & Tellegen, A. (2005). Developmental cascades: Linking academic achievement and externalizing and internalizing symptoms over 20 years. Developmental Psychology, 41, 733–746. 16173871 10.1037/0012-1649.41.5.733

[jcpp12655-bib-0031] Moffitt, T.E. (1993). Adolescence‐limited and life‐course‐persistent antisocial behavior: A developmental taxonomy. Psychological Review, 100, 674–701. 8255953

[jcpp12655-bib-0032] Morgan, A.B. , & Lilienfeld, S.O. (2000). A meta‐analytic review of the relation between antisocial behavior and neuropsychological measures of executive function. Clinical Psychology Review, 20, 113–136. 10660831 10.1016/s0272-7358(98)00096-8

[jcpp12655-bib-0034] Newsome, J. , Boisvert, D. , & Wright, J.P. (2014). Genetic and environmental influences on the co‐occurrence of early academic achievement and externalizing behavior. Journal of Criminal Justice, 42, 45–53.

[jcpp12655-bib-0035] Oliver, B. , & Plomin, R. (2007). Twins’ Early Development Study (TEDS): A multivariate, longitudinal genetic investigation of language, cognition and behavior problems from childhood through adolescence. Twin Research and Human Genetics, 10, 96–105. 17539369 10.1375/twin.10.1.96

[jcpp12655-bib-0036] Plomin, R. , DeFries, J.C. , Knopik, V.S. , & Neiderhiser, J.M. (2013). Behavioral genetics (6th edn). New York: Worth.

[jcpp12655-bib-0037] Price, T.S. , Freeman, B. , Craig, I.W. , Petrill, S.A. , Ebersole, L. , & Plomin, R. (2000). Infant zygosity can be assigned by parental report questionnaire data. Twin Research, 3, 129–133. 11035484 10.1375/136905200320565391

[jcpp12655-bib-0038] R Development Core Team (2015). R: A language and environment for statistical computing. Vienna: R Foundation for Statistical Computing Available from: http://www.R-project.org.

[jcpp12655-bib-0039] Reijneveld, S.A. , Brugman, E. , Verhulst, F.C. , & Verloove‐Vanhorick, S.P. (2005). Area deprivation and child psychosocial problems. Social Psychiatry and Psychiatric Epidemiology, 40, 18–23. 15624070 10.1007/s00127-005-0850-0

[jcpp12655-bib-0041] Roelofs, J. , Meesters, C. , ter Huurne, M. , Bamelis, L. , & Muris, P. (2006). On the links between attachment style, parental rearing behaviors, and internalizing and externalizing problems in non‐clinical children. Journal of Child and Family Studies, 15, 319–332.

[jcpp12655-bib-0042] Saudino, K.J. , & Plomin, R. (2007). Why are hyperactivity and academic achievement related? Child Development, 78, 972–986. 17517016 10.1111/j.1467-8624.2007.01044.x PMC4106298

[jcpp12655-bib-0043] Sewell, W. , & Hauser, R. (1975). Education, occupation, and earnings: Achievement in the early career. New York: Academic Press.

[jcpp12655-bib-0044] Shakeshaft, N.G. , Trzaskowski, M. , McMillan, A. , Rimfeld, K. , Krapohl, E. , Haworth, C.M. , … & Plomin, R. (2013). Strong genetic influence on a UK nationwide test of educational achievement at the end of compulsory education at age 16. PLoS ONE, 8, e80341. 24349000 10.1371/journal.pone.0080341 PMC3859476

[jcpp12655-bib-0045] Silk, J.S. , Steinberg, L. , & Morris, A.S. (2003). Adolescents’ emotion regulation in daily life: Links to depressive symptoms and problem behavior. Child Development, 74, 1869–1880. 14669901 10.1046/j.1467-8624.2003.00643.x

[jcpp12655-bib-0046] Steinberg, L. (2007). Risk taking in adolescence new perspectives from brain and behavioral science. Current Directions in Psychological Science, 16, 55–59.

[jcpp12655-bib-0047] Stone, L.L. , Otten, R. , Engels, R.C. , Vermulst, A.A. , & Janssens, J.M. (2010). Psychometric properties of the parent and teacher versions of the Strengths and Difficulties Questionnaire. Clinical Child and Family Psychology Review, 13, 254–274. 20589428 10.1007/s10567-010-0071-2 PMC2919684

[jcpp12655-bib-0048] Turkheimer, E. , & Waldron, M. (2000). Nonshared environment: A theoretical, methodological, and quantitative review. Psychological Bulletin, 126, 78–108. 10668351 10.1037/0033-2909.126.1.78

[jcpp12655-bib-0049] van Lier, P.A. , Vitaro, F. , Barker, E.D. , Brendgen, M. , Tremblay, R.E. , & Boivin, M. (2012). Peer victimization, poor academic achievement, and the link between childhood externalizing and internalizing problems. Child Development, 83, 1775–1788. 22716904 10.1111/j.1467-8624.2012.01802.x

[jcpp12655-bib-0050] Veldman, K. , Bültmann, U. , Stewart, R.E. , Ormel, J. , Verhulst, F.C. , & Reijneveld, S.A. (2014). Mental health problems and educational attainment in adolescence: 9‐year follow‐up of the TRAILS study. PLoS ONE, 9, e115070. 10.1371/journal.pone.0101751 PMC4105412 25047692

[jcpp12655-bib-0051] Vitaro, F. , Brendgen, M. , Larose, S. , & Trembaly, R.E. (2005). Kindergarten disruptive behaviors, protective factors, and educational achievement by early adulthood. Journal of Educational Psychology, 97, 617–629.

