# Supplementary material for: Externalizing problems in childhood and adolescence predict subsequent educational achievement but for different genetic and environmental reasons
Source: J Child Psychol Psychiatry. 2016 Nov 10;58(3):292–304. doi: 10.1111/jcpp.12655 (PMC5324692; doi:10.1111/jcpp.12655)
Supplement: Supplementary file 1 — Figure S1. Independent pathway model for conduct problems (age 4), hyperactivity (age 4), and educational achievement (age 16). Figure S2. Modified independent pathway model for peer problems (age 4), anxiety (age 4), conduct problems (age 4), and educational achievement (age 16). Table S1. Number of complete twin pairs across zygosity and sex for all study measures. Table S2. Phenotypic correlations between SDQ variables and educational achievement. Table S3. Cross‐sectional twin analysis results for SDQ variables. Table S4. Correlations between SDQ variables and educational achievement for MZ twin pairs. Table S5. Correlations between SDQ variables and educational achievement for DZ twin pairs. [file JCPP-58-292-s001.docx]

**Supporting online information for *Externalizing problems in childhood and adolescence predict subsequent educational achievement but for different genetic and environmental reasons* by Lewis, Asbury, and Plomin**

Table S1. Number of complete twin pairs across zygosity and sex for all study measures.

|  | Anxiety | Conduct | Hyper | Peers | Prosoc | Education |
| --- | --- | --- | --- | --- | --- | --- |
| *Age 4* |  |  |  |  |  |  |
| MZM | 1165 | 1166 | 1166 | 1163 | 1166 | - |
| MZF | 1350 | 1350 | 1348 | 1348 | 1350 | - |
| DZM | 1193 | 1196 | 1195 | 1193 | 1196 | - |
| DZF | 1247 | 1247 | 1245 | 1245 | 1247 | - |
| DZOS | 2344 | 2350 | 2345 | 2345 | 2352 | - |
| *Age 12* |  |  |  |  |  |  |
| MZM | 912 | 911 | 912 | 912 | 913 | - |
| MZF | 1130 | 1130 | 1130 | 1130 | 1132 | - |
| DZM | 844 | 845 | 845 | 845 | 847 | - |
| DZF | 968 | 968 | 968 | 968 | 973 | - |
| DZOS | 1742 | 1742 | 1741 | 1741 | 1744 | - |
| *Age 16* |  |  |  |  |  |  |
| MZM | - | 722 | 720 | - | 721 | 1012 |
| MZF | - | 1029 | 1028 | - | 1028 | 1253 |
| DZM | - | 670 | 670 | - | 670 | 948 |
| DZF | - | 889 | 886 | - | 888 | 1059 |
| DZOS | - | 1517 | 1513 | - | 1514 | 1991 |

Note. MZ = monozygotic; DZ = dizygotic; M = male; F = female; OS = opposite –sex; conduct = conduct problems; hyper = hyperactivity; peers = peer problems; prosoc = prosociality; education = educational achievement

Table S2. Phenotypic correlations between SDQ variables and educational achievement

|  | 1. | 2. | 3. | 4. | 5. | 6. | 7. | 8. | 9. | 10. | 11. | 12. | 13. |
| --- | --- | --- | --- | --- | --- | --- | --- | --- | --- | --- | --- | --- | --- |
| 1. Anxiety_Age4_ | - |  |  |  |  |  |  |  |  |  |  |  |  |
| 2. Conduct_Age4_ | .26 | - |  |  |  |  |  |  |  |  |  |  |  |
| 3. Hyper_Age4_ | .18 | .42 | - |  |  |  |  |  |  |  |  |  |  |
| 4. Peers_Age4_ | .29 | .23 | .14 | - |  |  |  |  |  |  |  |  |  |
| 5. Prosoc_Age4_ | -.05 | -.31 | -.24 | -.22 | - |  |  |  |  |  |  |  |  |
| 6. Anxiety_Age12_ | .31 | .18 | .12 | .17 | -.04 | - |  |  |  |  |  |  |  |
| 7. Conduct_Age12_ | .17 | .40 | .25 | .14 | -.14 | .31 | - |  |  |  |  |  |  |
| 8. Hyper_Age12_ | .14 | .31 | .44 | .16 | -.15 | .26 | .47 | - |  |  |  |  |  |
| 9. Peers_Age12_ | .16 | .18 | .15 | .24 | -.08 | .36 | .31 | .28 | - |  |  |  |  |
| 10. Prosoc_Age12_ | -.06 | -.16 | -.13 | -.18 | .33 | -.07 | -.31 | -.26 | -.18 | - |  |  |  |
| 11. Conduct_Age16_ | .11 | .29 | .19 | .10 | -.09 | .20 | .49 | .33 | .19 | -.20 | - |  |  |
| 12. Hyper_Age16_ | .14 | .25 | .32 | .15 | -.10 | .20 | .38 | .55 | .20 | -.189 | .53 | - |  |
| 13. Prosoc_Age16_ | -.04 | -.17 | -.12 | -.16 | .27 | -.09 | -.27 | -.22 | -.15 | .45 | -.40 | -.33 | - |
| 14. Education | -.06 | -.19 | -.23 | -.09 | .02 | -.10 | -.24 | -.35 | -.14 | .06 | -.27 | -.41 | .11 |

Note. Correlations are taken from one individual in each twin pair; conduct = conduct problems; hyper = hyperactivity; peers = peer problems; prosoc = prosociality; education = educational achievement; all coefficient’s > .03 are p < .01; n_range_ = 3854-7382.

Table S3. Cross-sectional twin analysis results for SDQ variables

| Variable | A | C | E |
| --- | --- | --- | --- |
| Anxiety_Age4_ | **.58** | .00 | **.42** |
| Conduct problems_Age4_ | **.64** | .00 | **.36** |
| Hyperactivity_Age4_ | **.42** | .00 | **.58** |
| Peer problems_Age4_ | **.67** | .01 | **.31** |
| Prosociality_Age4_ | **.59** | .00 | **.41** |
| Anxiety_Age12_ | **.53** | .05 | **.41** |
| Conduct problems_Age12_ | **.59** | **.18** | **.23** |
| Hyperactivity_Age12_ | **.74** | .00 | **.25** |
| Peer problems_Age12_ | **.72** | .00 | **.27** |
| Prosociality_Age12_ | **.64** | .12 | **.24** |
| Conduct problems_Age16_ | **.74** | .00 | **.27** |
| Hyperactivity_Age16_ | **.74** | .00 | **.26** |
| Prosociality_Age16_ | **.53** | **.32** | **.15** |

Note. A = additive genetic effects; C = shared-environment effects; E = nonshared-environment effects; bolded = p < .05.

Table S4. Correlations between SDQ variables and educational achievement for MZ twin pairs

|  | 1. | 2. | 3. | 4. | 5. | 6. | 7. | 8. | 9. | 10. | 11. | 12. | 13. | 14. |
| --- | --- | --- | --- | --- | --- | --- | --- | --- | --- | --- | --- | --- | --- | --- |
| 1. Anxiety_Age4_ | .56 |  |  |  |  |  |  |  |  |  |  |  |  |  |
| 2. Conduct_Age4_ | .26 | .64 |  |  |  |  |  |  |  |  |  |  |  |  |
| 3. Hyper_Age4_ | .21 | .33 | .52 |  |  |  |  |  |  |  |  |  |  |  |
| 4. Peers_Age4_ | .19 | .21 | .22 | .65 |  |  |  |  |  |  |  |  |  |  |
| 5. Prosoc_Age4_ | -.05 | -.15 | -.17 | -.20 | .59 |  |  |  |  |  |  |  |  |  |
| 6. Anxiety_Age12_ | .26 | .16 | .10 | .10 | -.02 | .59 |  |  |  |  |  |  |  |  |
| 7. Conduct_Age12_ | .15 | .36 | .23 | .12 | -.12 | .30 | .76 |  |  |  |  |  |  |  |
| 8. Hyper_Age12_ | .15 | .30 | .33 | .17 | -.14 | .26 | .45 | .75 |  |  |  |  |  |  |
| 9. Peers_Age12_ | .11 | .21 | .16 | .17 | -.07 | .29 | .27 | .28 | .70 |  |  |  |  |  |
| 10. Prosoc_Age12_ | -.05 | -.14 | -.15 | -.14 | .27 | -.13 | -.28 | -.29 | -.20 | .77 |  |  |  |  |
| 11. Conduct_Age16_ | .11 | .25 | .19 | .12 | -.09 | .17 | .37 | .32 | .14 | -.17 | .71 |  |  |  |
| 12. Hyper_Age16_ | .12 | .22 | .24 | .14 | -.12 | .17 | .36 | .46 | .18 | -.19 | .42 | .73 |  |  |
| 13. Prosoc_Age16_ | -.04 | -.16 | -.18 | -.17 | .25 | -.10 | -.30 | -.30 | -.15 | .43 | -.35 | -.35 | .85 |  |
| 14. Education | -.07 | -.20 | -.20 | -.07 | .03 | -.10 | -.24 | -.32 | -.15 | .10 | -.23 | -.35 | .10 | .89 |

Note. conduct = conduct problems; hyper = hyperactivity; peers = peer problems; prosoc = prosociality; education = educational achievement; all coefficient’s > .07 are p < .01; n_range_ = 1419-2516.

Table S5. Correlations between SDQ variables and educational achievement for DZ twin pairs

|  | 1. | 2. | 3. | 4. | 5. | 6. | 7. | 8. | 9. | 10. | 11. | 12. | 13. | 14. |
| --- | --- | --- | --- | --- | --- | --- | --- | --- | --- | --- | --- | --- | --- | --- |
| 1. Anxiety_Age4_ | .31 |  |  |  |  |  |  |  |  |  |  |  |  |  |
| 2. Conduct_Age4_ | .22 | .34 |  |  |  |  |  |  |  |  |  |  |  |  |
| 3. Hyper_Age4_ | .16 | .16 | -.05 |  |  |  |  |  |  |  |  |  |  |  |
| 4. Peers_Age4_ | .16 | .23 | .17 | .36 |  |  |  |  |  |  |  |  |  |  |
| 5. Prosoc_Age4_ | -.04 | -.06 | -.07 | -.12 | .32 |  |  |  |  |  |  |  |  |  |
| 6. Anxiety_Age12_ | .19 | .15 | .09 | .13 | -.05 | .33 |  |  |  |  |  |  |  |  |
| 7. Conduct_Age12_ | .16 | .24 | .12 | .17 | -.09 | .22 | .50 |  |  |  |  |  |  |  |
| 8. Hyper_Age12_ | .13 | .17 | .05 | .16 | -.08 | .22 | .23 | .23 |  |  |  |  |  |  |
| 9. Peers_Age12_ | .16 | .15 | .07 | .18 | -.05 | .20 | .24 | .18 | .38 |  |  |  |  |  |
| 10. Prosoc_Age12_ | -.06 | -.12 | -.12 | -.12 | .17 | -.10 | -.21 | -.21 | -.16 | .46 |  |  |  |  |
| 11. Conduct_Age16_ | .11 | .14 | .09 | .10 | -.03 | .15 | .20 | .12 | .13 | -.11 | .38 |  |  |  |
| 12. Hyper_Age16_ | .09 | .13 | .06 | .11 | -.04 | .14 | .16 | .10 | .17 | -.15 | .23 | .25 |  |  |
| 13. Prosoc_Age16_ | -.05 | -.09 | -.07 | -.11 | .14 | -.09 | -.17 | -.15 | -.09 | .22 | -.20 | -.23 | .58 |  |
| 14. Education | -.08 | -.13 | -.07 | -.09 | .01 | -.08 | -.18 | -.13 | -.13 | .05 | -.18 | -.17 | .10 | .58 |

Note. conduct = conduct problems; hyper = hyperactivity; peers = peer problems; prosoc = prosociality; education = educational achievement; all coefficient’s > .05 or < -.05 are p < .01; n_range_ = 2433-4795.

Figure S1. Independent pathway model for conduct problems (age 4), hyperactivity (age 4), and educational achievement (age 16).


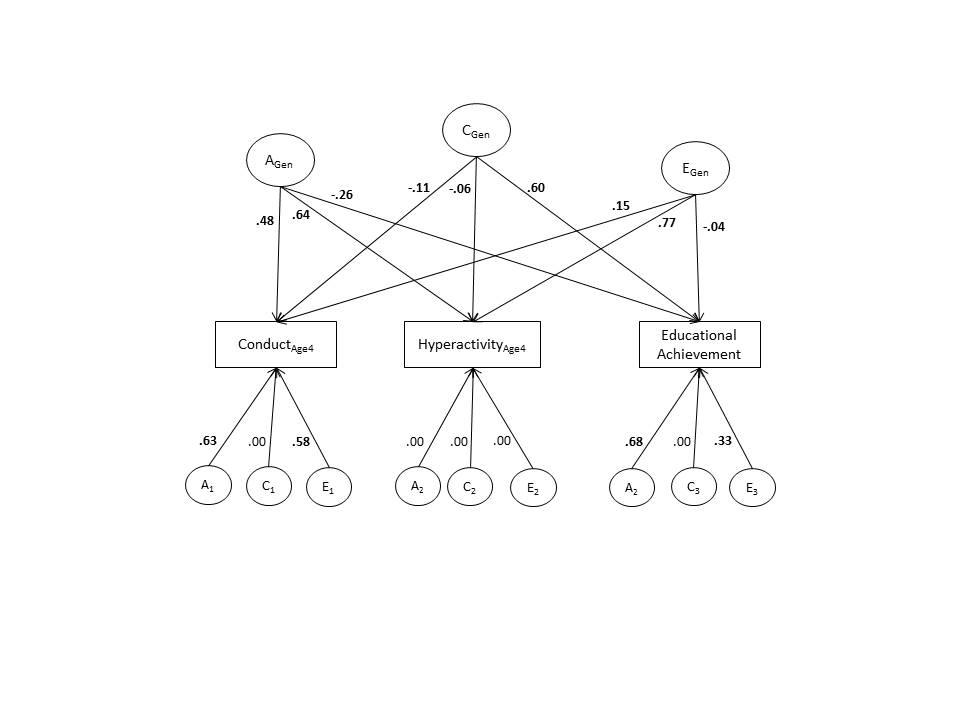


Note. A = additive genetic effects; C = shared-environment effects; E = nonshared-environment effects; gene = general factor; bolded = p < .05.

Figure S2. Modified independent pathway model for peer problems (age 4), anxiety (age 4), conduct problems (age 4), and educational achievement (age 16).


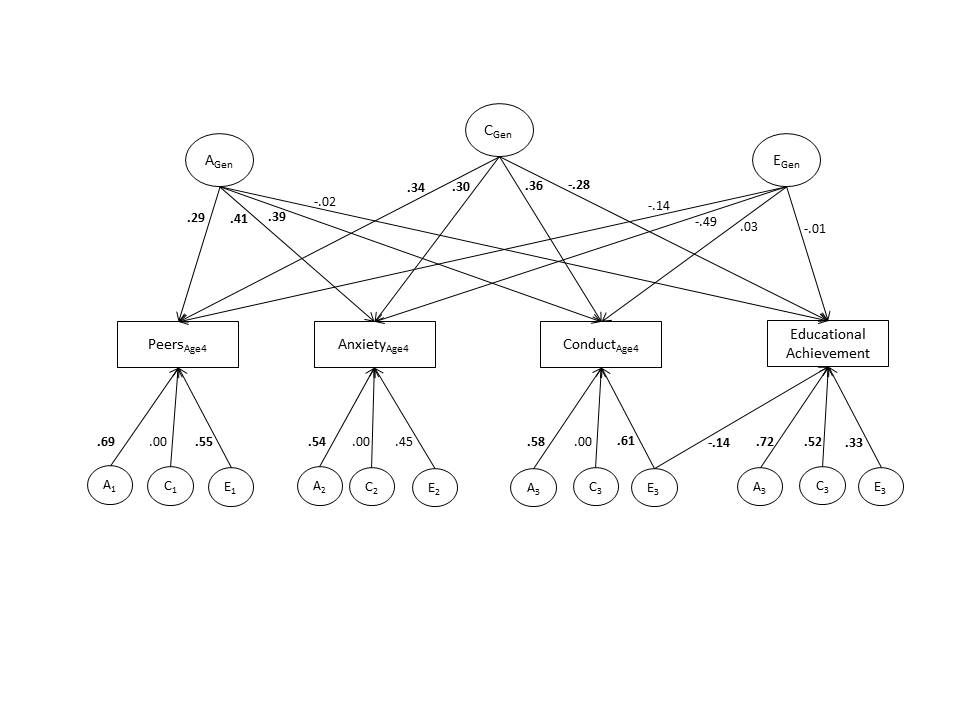


Note. A = additive genetic effects; C = shared-environment effects; E = nonshared-environment effects; gene = general factor; bolded = p < .05.
